# What Is Medical Responsibility?

**Published:** 1916-06-24

**Authors:** 


### The Hospital

The Workers' Newspaper of Administrative Medicine and Institutional Life, Administration, National Insurance and Health.

No. 1567, Vol. LX. SATURDAY, JUNE 24, 1916. PRICE ONE PENNY.

### WHAT IS MEDICAL RESPONSIBILITY?

This is a question which, at least when it concerns the individual patient, the plain man will answer without difficulty. The doctor is in charge of the patient and is answerable for his welfare. Both for what is done and for what is left undone the medical adviser must accept responsibility. It is for him both to give orders and to see that they are obeyed, and if his authority is neglected or challenged he has no option but to retire from the case. What amount of attention and supervision is required is part of the medical judgment of the position, and here, as in other directions, command must be absolute. In a word, as responsibility is all-embracing so also must be authority. Further, successfully to carry this responsibility and authority it is necessary to have suffered the discipline of the medical curriculum, together with such further experiences as come within reach. Such at least is the view upon which rests the modern organisation of medical education and practice. There is no half-way house. Both according to the demands of the law and according to common sense a given individual either is or is not a qualified medical practitioner. It follows therefore that as judged by these standards medical responsibility can only be accepted by one who has satisfied at least the minimum requirements of a medical training. There is one very impressive illustration of the truth of this statement in the domestic regulations of the medical profession itself. Until a comparatively few years ago there existed in the profession a group of men who were known as unqualified assistants. Many of these had acquired much practical experience, but they had failed to satisfy either the demands of the curriculum or the demands of the examining bodies. No doubt the employment of such an assistant was often a convenience to a practitioner. He could act as a substitute for the doctor in what appeared to be non-serious cases, and could undertake many proceedings with a fair degree of satisfaction and success. Yet the dangers and risks of such an arrangement were great, and the consideration of these led to the formal suppression of the unqualified assistant by the summary action of the General Medical Council. Apart from the abuses attending his existence he was an anomaly and a contradiction, for the root of the matter was not in him. He had never received the training affirmed and accepted as a necessary qualification for the adequate discharge of medical responsibility. This responsibility as it knows no half-measures in its discharge knows equally no half-measures in its preparation.

With such a theory and practice in its own household the medical profession must necessarily keep a watchful and even a jealous eye on enterprises taking origin without its bounds. That any given individual is free either to give or to accept advice on questions of health or of disease is not in dispute. Such is the law of the land, and no one proposes to challenge it. But as the profession has expelled the unqualified assistant from its own household—acting in this matter not from interest but according to a well-defined principle—it can hardly be expected to permit a similar institution to be thrust upon it from the outside. And in view of the special responsibility of the General Medical Council in this matter the Council may be expected to see that any such attempt is frustrated.

These general considerations are appropriate at this particular moment in consequence of a scheme recently suggested for the care of many children suffering from measles. The compulsory notification of cases of measles has made officially manifest, what indeed has long been popularly known, that many children attacked by this disease either receive no medical care or receive such care only when dangerous or alarming symptoms appear. To rectify such a state of matters is a philanthropic ambition, and, especially in view of the drainage of war on the population, the proposal may be recognised as a patriotic one. The neglect which it is hoped to amend is without question responsible both for suffering and for the loss of lives which under a kinder fate would have rendered service to the nation. But if the thing is to be done satisfactorily it must be done thoroughly. If it is a national interest and a national duty to provide these children with medical care, such care must be real and substantial; and the country which is to get the benefit of it must meet the necessary expense. But the proposals which have been advanced do not seem to satisfy these conditions. The suggestion is that these children shall be attended not by doctors but by nurses, so long, that is, as the nurse does not consider the intervention of a medical practitioner to be necessary. No doubt she is to be provided with certain printed rules which profess a definition of the circumstances in which medical intervention is officially declared to be essential; and she is in a pious and general fashion to advise parents to consult a doctor. But so long as things in her judgment are running smoothly and none of the events officially declared to be alarming arrives, she may "do her best for the patient." Even so, in former days, the medical practitioner directed the "unqualified assistant," who was always understood to send for his chief when matters became anxious or critical. The principle in the two cases is the same and reposes on the notion that mild cases of illness may be safely conducted by an unqualified person, who, further, subject to instructions, written or oral, may be trusted to judge when the qualified practitioner is necessary. The unqualified assistant in his frank and open form has been declared inadmissible, and a specious revival of the arrangement cannot be permitted even though it is philanthropy which makes the proposition. Admittedly it is an unhappy state of matters that half, or more than half, of the cases of measles among the poorer classes are not seen by a doctor. But if this defect is to be remedied for humanitarian and national ends the remedy must be a real one. To send an unqualified person with instructions to take no medical responsibility but to "do the best for the patient" is a contradiction in terms. It means in substance treatment of the patient, and whatever the responsibility be called it is essentially one which can only be assumed with safety by a qualified medical practitioner.

